# Environmental impacts on diapause and survival of the alfalfa leafcutting bee, *Megachile rotundata*

**DOI:** 10.1371/journal.pone.0254651

**Published:** 2021-08-03

**Authors:** Elisabeth S. Wilson, Claire E. Murphy, Covey Wong, Joseph P. Rinehart, George D. Yocum, Julia H. Bowsher

**Affiliations:** 1 Department of Biological Sciences, North Dakota State University, Fargo, ND, United States of America; 2 Biology Department, William & Mary, Williamsburg, VA, United States of America; 3 Bioscience Research Laboratory, U.S. Department of Agriculture/Agricultural Research Station, Fargo, ND, United States of America; University of Cincinnati, UNITED STATES

## Abstract

*Megachile rotundata* exhibits a facultative prepupal diapause but the cues regulating diapause initiation are not well understood. Possible cues include daylength and temperature. *Megachile rotundata* females experience changing daylengths over the nesting season that may influence diapause incidence in their offspring through a maternal effect. Juvenile *M*. *rotundata* spend their developmental period confined in a nesting cavity, potentially subjected to stressful temperatures that may affect diapause incidence and survival. To estimate the impact of daylength and nest cavity temperature on offspring diapause, we designed a 3D printed box with iButtons that measured nest cavity temperature. We observed nest building throughout the season, monitored nest cavity temperature, and followed offspring through development to measure diapause incidence and mortality. We found that daylength was a cue for diapause, and nest cavity temperature did not influence diapause incidence. Eggs laid during long days had a lower probability of diapause. Siblings tended to have the same diapause status, explaining a lot of the remaining variance in diapause incidence. Some females established nests that contained both diapausing and nondiapausing individuals, which were distributed throughout the nest. Nest cavities reached stressful temperatures, which decreased survival. Mortality was significantly higher in nondiapausing bees and the individuals that were laid first in the nest. In conclusion, we demonstrate a maternal effect for diapause that is mediated by daylength and is independent of nest box temperature.

## 1. Introduction

Diapause allows insects to avoid seasonally reoccurring stressors such as those associated with temperate winters and allows for synchronization of life histories with optimal resources such as flower bloom [1]. Diapause may be either obligate or facultative, with the cues triggering facultative diapause varying across species [2]. Many insects with facultative diapause have a critical daylength that programs diapause [2] and diapause initiation can also be influenced by temperature [3–5]. The Mediterranean tiger moth *Cymbalophora pudica* relies on daylength as a cue for diapause length, with long daylengths shortening diapause and short daylengths lengthening diapause [6]. The blow fly *Calliphora vicina* produces fewer diapausing offspring at 20˚C than at 15˚C [3]. How these cues affect diapause can be complex. For pollinating insects, understanding the underlying mechanisms of diapause initiation is critical to management of agricultural bee populations because rising global temperatures have the potential to increase the proportion of nondiapausing bees [5]. Nondiapausing bees lowers yield of offspring because the alfalfa season is not long enough to accommodate the full second generation [5] and nondiapausing bees have the potential to kill nest mates when they emerge [7].

*Megachile rotundata*, the alfalfa leafcutting bee, is a solitary bee used extensively in the production of alfalfa seed [8]. *Megachile rotundata* adults emerge in the summer, mate, and females build linear nests made of individual cells of leaves and provision them with pollen and nectar [8]. Once a brood cell is complete, the female will lay an egg on top of the provision, seal the cell with additional leaf pieces, and then begin the next cell. Once the nest is complete the female will seal off the nest with a cap. Eggs will complete embryogenesis in approximately 2–3 days and continue to develop through five instars [9]. *Megachile rotundata* has a facultative diapause with individuals either entering diapause in the pre-pupa stage or skipping diapause to emerge in the same summer as the parent generation, with the following generation then entering diapause [10].

In *M*. *rotundata*, the leading hypothesis is that diapause is under maternal control [10], although the exact mechanism causing nondiapausing individuals remains unclear. Several studies have noted environmental factors that may influence *M*. *rotundata* diapause incidence including length of the day [11], warm temperatures [4, 5, 10], and the amount of food provision [12]. Several field studies have provided evidence that daylength is important to diapause determination in *M*. *rotundata*. Nondiapausing individuals are often offspring from the first nests completed during the summer when daylengths are long. In the United States, nondiapausing individuals may comprise 34–54% of the brood cells in early July, but fall to percentages in the single digits by the end of July or early August when daylength decreases [4, 5, 13]. However, Canadian populations only reach up to 5% of nondiapausing individuals [14]. These consistent differences in diapause incidence across latitude suggest that mothers respond to increasing daylength by laying nondiapausing eggs. However, mothers experimentally exposed to longer days did not necessarily have higher rates of nondiapausing offspring [11] indicating daylength may not be the sole cue for diapause incidence.

While many studies have noted the importance of daylength, temperature may also impact diapause initiation. *Megachile rotundata* are likely to be impacted by heat stress in the nesting box with temperatures reaching above 40˚C [15]. Heat stress has been shown to decrease development time and lead to higher rates of nondiapausing individuals [4, 5, 16]. Furthermore, several studies have noted that temperature stress may also cause bees that have already initiated diapause to avert to a nondiapausing state and emerge in the same year [4, 5]. Therefore, heat stress could increase the frequency of nondiapausing individuals by both lowering rates of diapause incidence and by reversing diapause in bees that have already initiated diapause. Temperature could also influence diapause through a growing degree day model in which increased degree days reduces diapause incidence.

Our understanding of diapause determination in *M*. *rotundata* is further complicated by the possibility of diapausing and nondiapausing individuals within the same nest. The perceived general rule is that *M*. *rotundata* nests contain only diapausing or nondiapausing progeny [13], but there are reports of mixed nests which contain both diapausing and nondiapausing individuals [7, 14]. Tepedino and Frohlich [7] observed that in mixed nests the nondiapausing bees were female biased and frequently found behind their diapausing siblings. Within mixed nests the nondiapausing progeny were normally grouped in consecutive cocoons [14]. Mothers build brood cells sequentially at the rate of approximately one brood cells per day [17, 18] starting at the back of the nest. Nests containing both diapausing and nondiapausing progeny creates the potential for fratricide when a nondiapausing individual must chew through its diapausing siblings to emerge [7]. The existence of mixed nests suggest that diapause incidence might be influenced by environmental factors that are beyond maternal control.

Our goal was to test the relative contributions of daylength, nest cavity temperature, and position within the nest to diapause status in *M*. *rotundata*. We hypothesized that the daylength the mother experienced while laying an egg would influence diapause initiation in the offspring. We also hypothesized nest cavity temperature during larval development would influence diapause initiation. We measured the effect of temperature on diapause incidence by monitoring cavity temperature in the field. The date a nest was completed was used to calculate the daylength on the day each egg was laid, and nests were x-rayed to determine diapause status and brood cell position. We also investigated whether high nest temperatures increased mortality. Our results indicate that daylength, not temperature, influences diapause incidence in *M*. *rotundata* under our field conditions.

## 2. Methods

This is a continuation of a previously published study that investigated the effects of temperature on maternal nesting choice using the same nesting boxes and field set up [15]. Additional details are provided in that publication.

### 2.1 Description of field site and nest boxes

**Fig 1:**
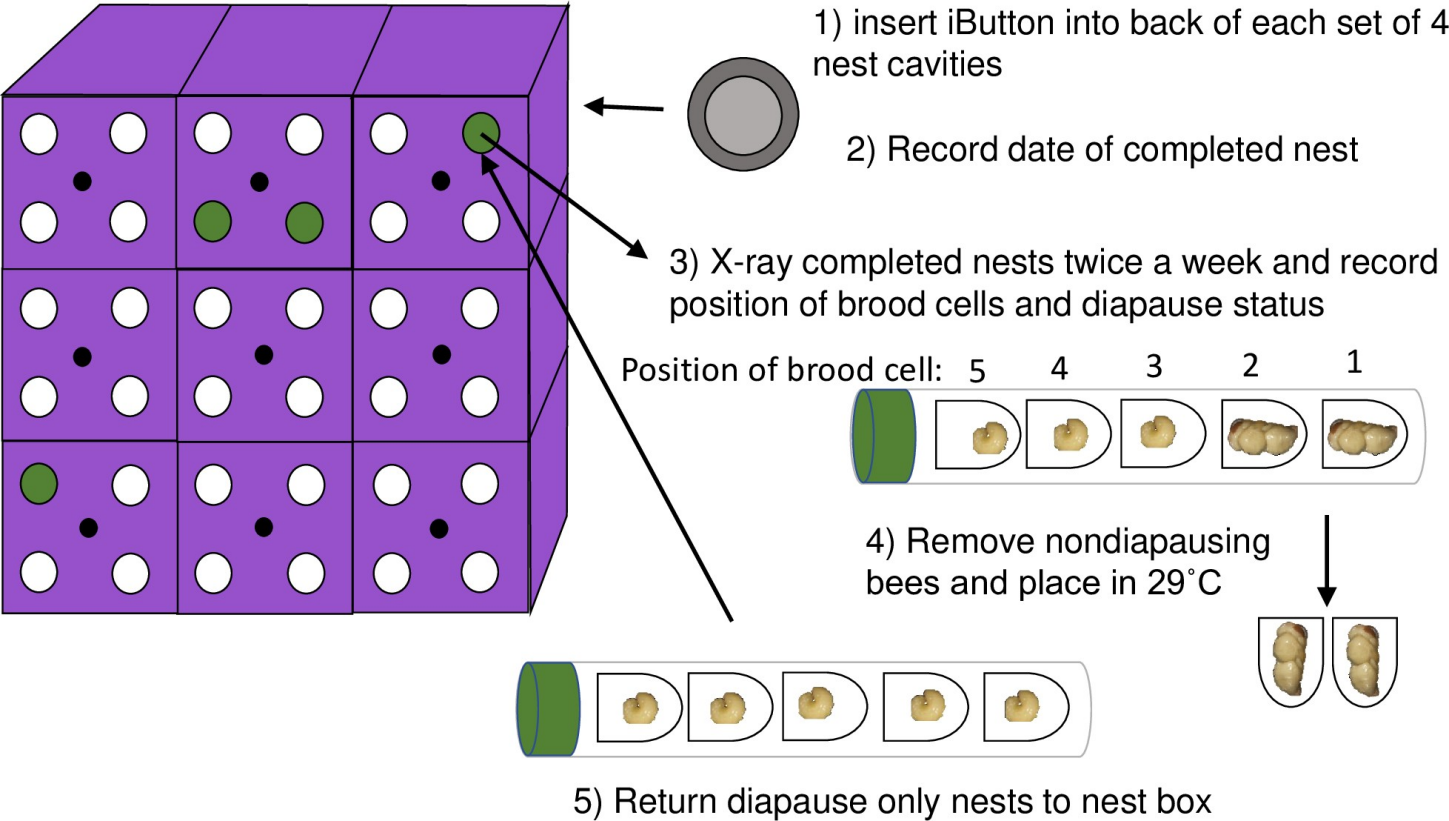
Experimental design. A nest box was constructed of 3D printed plastic blocks, each with four cavities. (1) The temperature of each block was recorded throughout the season using iButtons. (2) Nests were checked daily for completion. (3) Nests were X-rayed to determine diapause status, and (4) nondiapausing individuals were removed from the field and allowed to emerge in the lab. (5) Diapausing individuals were returned to the nest box and experienced field temperatures until September 22^nd^ (photo credit: Manitoba Department of Agriculture).

Three nest boxes were placed 200 m apart between the edge of an alfalfa field and a drainage ditch containing multiple forbs in Fargo, North Dakota, USA (46˚55’15” N, 96˚51’17” W). Each nest box consisted of 36 3D printed blocks (measuring 60mm x 60mm x 82mm), each of which contained four equally spaced nesting cavities (lined with a paper straw for easy nest removal) on the front and a single cavity to accommodate an iButton temperature datalogger on the back that recorded temperature every 15 minutes (Fig 1). Each of the four sides of the nesting box were constructed by stacking nine blocks in a three by three pattern, resulting in 36 nesting cavities per side, and 144 cavities per nest box. Each nest box was oriented in the field using a compass so that the sides faced Northwest (NW), Northeast (NE), Southwest (SW), and Southeast (SE). Temperatures were recorded from each block from June 21^st^, 2018 through September 22, 2018. *Megachile rotundata* (JVM Leafcutters, Nampa, Idaho) were released at each nest box on June 20^th^ and June 26^th^ to ensure a large population of nesting females.

### 2.2 Monitoring nesting behavior and nest size

The nesting period extended from June 26^th^ to August 2^nd^ 2018 and boxes were checked daily for completed nests, indicated by the presence of a leaf cap. Completed nests were x-rayed (Faxitron Bioptics LLC, Tucson, AZ) twice weekly from July 6^th^ to August 2^nd^. After the nesting period ended, nests were x-rayed August 15^th^, September 3^rd^, and September 22^nd^. Nest parameters measured by x-ray included the number of brood cells, brood cell position, diapause state, and evidence of parasitism. Brood cell position was numbered starting from the first brood cell built by the mother to the final brood cell. The first brood cell was in the back of the cavity and the final brood cell positioned by the nest entrance. Nests contained up to nine brood cells, with the average being seven. Offspring that were identified as nondiapausing were dissected from the nest upon reaching the pupal stage and their position and date were recorded (Fig 1), after which they were placed in 24-well plates with lids in a 29˚C incubator in darkness and 75% humidity and allowed to continue to develop, with emergence date, and sex recorded. In mixed nests, diapausing offspring were discarded because the nest was destroyed when the nondiapausers were removed. Nests with only diapausing offspring remained in the field until Sept 22^nd^, after which they were also dissected from the nest and placed in 24-well plates with lids in a 6˚C incubator in darkness and 40% humidity for the remainder of the diapause period. Once the bees transitioned to post-diapause quiescence [19], the plates were placed in 29˚C on Dec. 11^th^ to initiate pupal development and adult emergence. Bees were considered emerged as adults when they had completely exited the brood cell. Emergence date and sex were recorded.

### 2.3 Data processing

iButtons were downloaded individually then combined and processed using R ([20], version 3.5.2) and RStudio ([21], version 1.1.419) with packages *lubridate* ([22], version 1.7.9.2), *tidyr* ([23], version 1.1.2), *stringr* ([24], version 1.4.0), and *dplyr* ([25], version 1.0.2).

The dates of the developmental period was estimated for each individual. The developmental period was defined as the number of days from egg laying to the point at which bees pupated, and was estimated from the data. The date the nest was completed, as determined by the presence of a leaf cap, was used to estimate the date each egg was laid by assuming the female built the nest at the rate of one brood cell per day [17, 18]. We then calculated the median number of days from the day the egg was laid to the day pupation was observed via X-ray for nondiapausing individuals. This median developmental period was 22 days. We used a 22-day interval to calculate temperature variables for each individual in the dataset. For example, the average temperature during the developmental period for each bee was calculated by averaging the iButton temperatures from the estimated date the egg was laid to 22 days later. We calculated the degree days over the same period using the *gdd* function in the *pollen* package ([26], version 0.72.0). The minimum temperature for development was set at 18˚C [27] and the upper limit at 35˚C [28]. We calculated time spent above 35˚C and 40˚C for each bee. These temperatures were chosen as biologically critical thresholds because at 35˚C *M*. *rotundata* start to produce HSP70 proteins, an indicator of thermal stress, and HSP70 proteins peak at 42˚C [28]. For the survival analysis, we calculated the time spent above 35˚C and 40˚C for each bee from the time the egg was laid to the date the nests were brought in from the field (Sept 22^nd^).

The daylength when each brood cell was built was calculated using the *geosphere* package ([29], version 1.5–10). The date each brood cell was built was estimated as described above. The daylength in hours was calculated for each day based on the latitude (46.909°N) of the field site. We calculated the change in daylength for each day by subtracting daylength from that of the previous day.

We observed females in the field tearing out existing nests to make their own. To eliminate those nests from the dataset, we counted the number of brood cells in the initial X-ray of each nest and compared that to the final X-ray taken at the end of the season. Nests that had a different number of brood cells from the beginning of the season to the end were removed from the dataset.

### 2.4 Diapause analysis

In order to test the relative influence of nest temperature and daylength on diapause incidence, we used generalized linear mixed effects models using the *lme4* package ([30], version 1.1–26) with p-values calculated using *lmerTest* ([31], version 3.1–3). The response variable for our model was diapause (1) or non-diapause (0) for each individual. The covariates were average temperature during the developmental period, degree days during the developmental period, daylength for the day the egg was laid, change in daylength between the day the egg was laid and the day before, and position of the brood cell in the nest with one designating the first brood cell built in the straw. All covariates were centered and scaled. Because the temperature variables were highly correlated, models were built that only included one of each of those variables, then compared for model fit using the package *MuMIn* ([32], version 1.43.17). The random effects were nest box replicate and maternal ID number. Maternal ID was included as a random effect to account for the fact that every individual within a nest shared the same nest environment, spent juvenile development during the same period of the season, and had the same mother. We tested a separate model that was the same in all respects except that change in daylength was substituted for daylength. The best model was determined using Akaike Information Criteria and model comparisons were preformed using ANOVA in the package *MuMIn* ([32], version 1.43.17). Residual diagnostics were preformed using *DHARMa* ([33], version 0.3.3.0). Data was graphed using *ggplot2* ([34], version 3.3.3).

### 2.5 Survival analysis

To understand what factors influenced survival, we used general linear mixed models using the same packages described above. Our response variable was emerged adults (1) and those that did not emerge (0). Covariates included whether the bees initiated diapause (1) or not (0), the position of the brood cell in the nest, and the daylength when the egg was laid. The temperature variables were calculated in the same way as the diapause analysis and included average temperature, the number of hours above 35˚C and the number of hours above 40˚C from the day the egg was laid until 22 days later. Temperature variables were tested by substituting them into the model because they were highly correlated. Covariates were centered and scaled. Random effects include replicate and maternal ID. Results were visualized with *ggplot2* ([34], version 3.3.3) and model predictions were graphed using *ggeffects* ([35], version 1.0.1)

## 3. Results

### 3.1 Nesting box temperatures and microclimates

**Fig 2:**
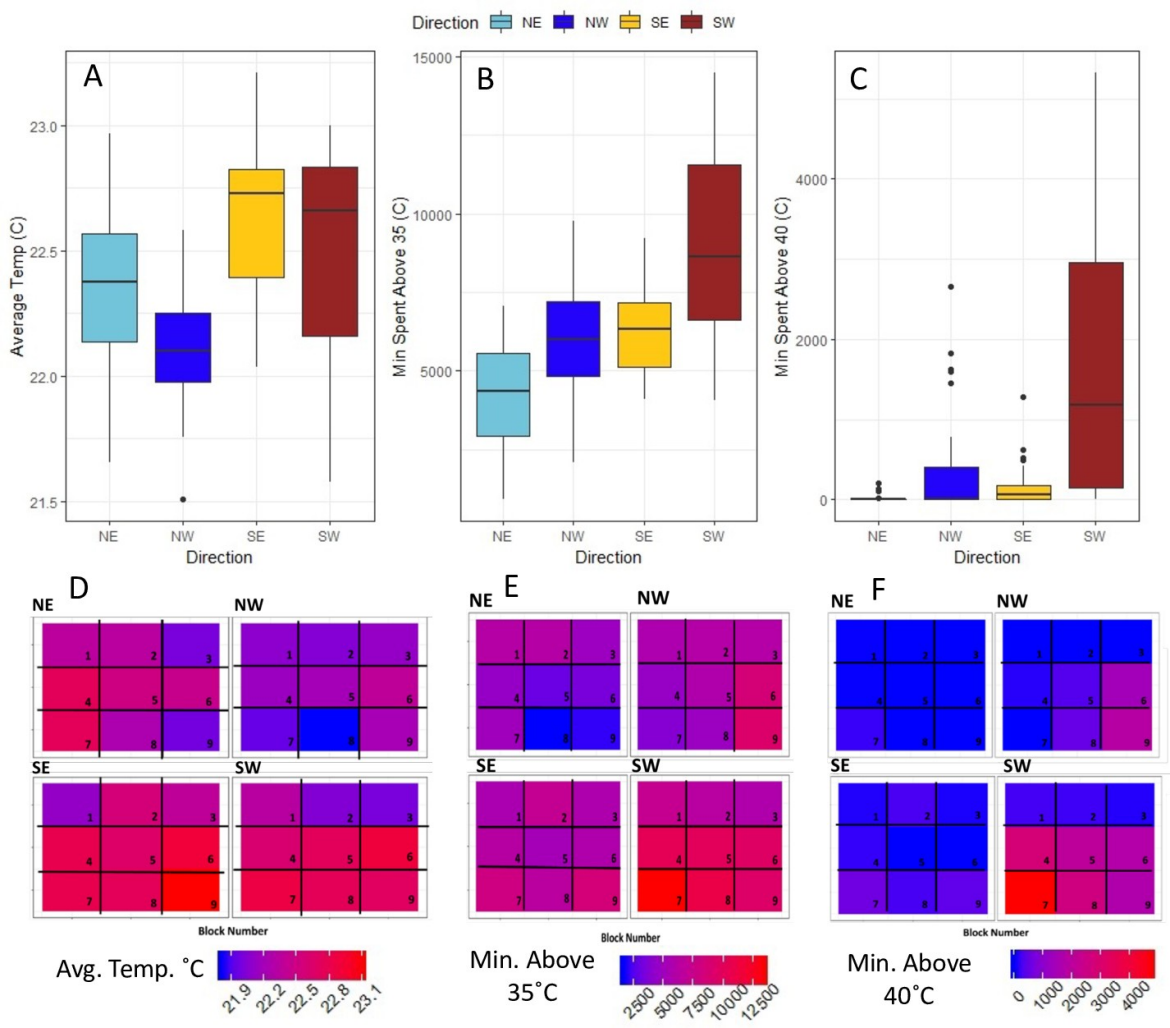
Nest cavity temperatures by the direction the nest cavity was facing. (A) Average temperature, (B) amount of time spent above 35˚C during the filed season, (C) amount of time spent above 40˚C during the field season. (D-F) Heat maps correspond to each direction the nest cavities were facing and each side divided into nine regions that correspond to the iButtons. (D) Average temperature, (E) amount of time spent above 35˚C during the filed season, and (F) amount of time spent above 40˚C during the field season.

Offspring were exposed to a highly variable range of temperatures in the nesting box throughout the season (Fig 2). The nesting boxes reached a minimum temperature of 4.5˚C and a maximum temperature of 48.5˚C. The NE reached a maximum temperature of 42˚C on June 28^th^, 2018. The NW reached a maximum temperature of 45.5˚C on Sept. 10^th^, 2018. The SE reached a maximum temperature of 44˚C on Aug. 16^th^ and the SW reached a maximum temperature of 48.5˚C on August 16^th^, and Sept. 10^th^, 2018.

Average cavity temperatures over the season were significantly impacted by direction the cavity faced and by the block location in the nest box (Fig 2A and 2D; Block: F_(8, 143)_ = 357.49, p<0.0001, Direction: F_(3,143)_ = 1074.49, p<0.0001, Fig 2A and 2D). The interaction between block and direction was also significant (F_(24, 143)_ = 119.54, p<0.0001). The model explained 72.75% of the variance in average cavity temperature when all variables were included. Time spent above 40˚C during the field season was also significantly impacted by block (Fig 2C, F_(8,143)_ = 396.16, p<0.0001), direction (Fig 2F, F_(3,143)_ = 2548.99, p<0.0001), and their interaction (F_(24,143)_ = 326.15, p<0.0001). Nest cavities that faced south spent the most time above 40˚C. The full model explained 84.78% of the variance in time spent above 40˚C. Like the other variables, time spent above 35˚C over the season was significantly impacted by both block (Fig 2B, F_(8,143)_ = 32.801, p = <0.0001), direction (Fig 2E, F_(3,143)_ = 2462.13, p<0.0001), and their interaction (F_(24,143)_ = 249.78, p<0.0001). The full model explained 81.08% of the variance in time spent above 35˚C.

### 3.2 Diapause initiation models

The diapause analysis included 1,594 individuals from 245 nests. The first nest was completed on June 23^rd^ and the last nest on August 15^th^. Most of the individuals initiated diapause (86.4%). The daylength during the nesting period ranged from 15.88 hours to 14.34 hours, average nest temperature ranged from 20.2°C to 26.3°C, and degree days ranged from 128.5 to 202.5. The sex ratio was female-biased for nondiapausing individuals (69.5%) and was significantly different from the sex ratio of diapausing individuals (39.23% female: χ^2^ = 47.753, df = 1, p<0.0001).

**Fig 3:**
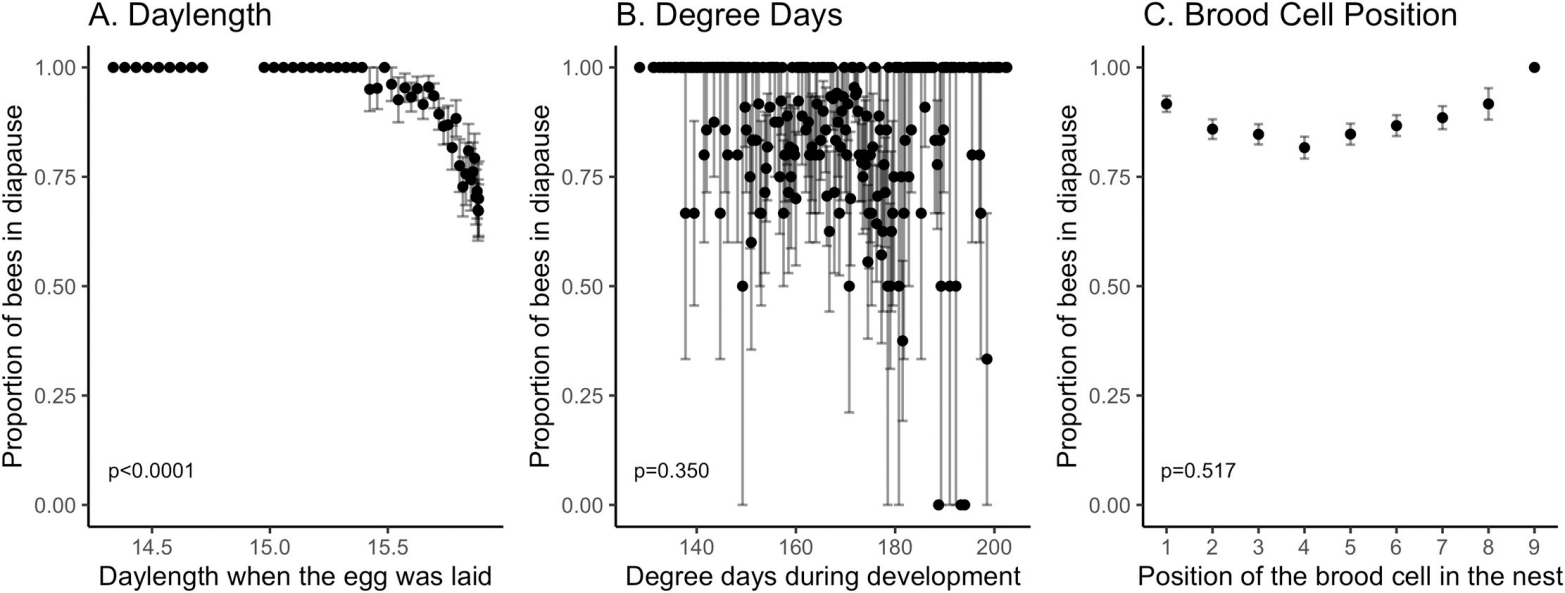
Diapause incidence. Daylength significantly influenced the rate of diapause (A). Neither degree days (B) nor brood cell position (C) influenced diapause incidence. In (C), position one is the first brood cell built in the nest. Whiskers denote the standard error of the mean.

Daylength and not temperature influenced diapause initiation. The best fit model included daylength (Fig 3A; Z_(1, 1594)_ = -4.140, p<0.0001) as a covariate and maternal ID as a random effect (variance = 24.68±4.968). Longer daylengths had a significantly lower probability of diapause (Fig 3A and 3D). The accumulated degree days over the developmental period did not significantly influence diapause incidence (Fig 3B; Z_(1, 1594)_ = 0.934, p = 0.35). The position of the brood cell in the nest was also not significant (Fig 3C; Z_(1, 1594)_ = -0.645, p = 0.52). We tested for multicollinearity between daylength, degree days and position of the brood cell to determine if these variables were influenced by each other. All variance inflation factors were below 2, indicating that the variables did not co-vary. The covariates explained 44.2% of the variation in diapause incidence. The random effect of maternal ID increased the R^2^ to 0.93. The model that included daylength was not significantly different from a model that included change in daylength (delta AIC = 4.2, χ^2^ = 0, df = 0, p = 1). We also tested additional temperature variables to determine if different aspects of temperature exposure besides degree days would influence diapause incidence. Because the temperature variables were highly correlated with each other, they were tested for significance by substituting into the model. We tested average temperature during the developmental period, which was the date the brood cell was laid until 22 days later (Z_(1, 1549)_ = 0.532, p = 0.60). We tested the number of hours spent above 33°C (Z_(1, 549)_ = 1.356, p = 0.18), above 35°C (Z_(1, 1594)_ = 0.634, p = 0.53), and above 40˚C (Z_(1, 1,549)_ = 0.1080, p = 0.28) during the developmental period. Like degree days, none of the other temperature variables were significantly influenced diapause incidence.

### 3.3 Nests with both diapausing and nondiapausing offspring

**Fig 4:**
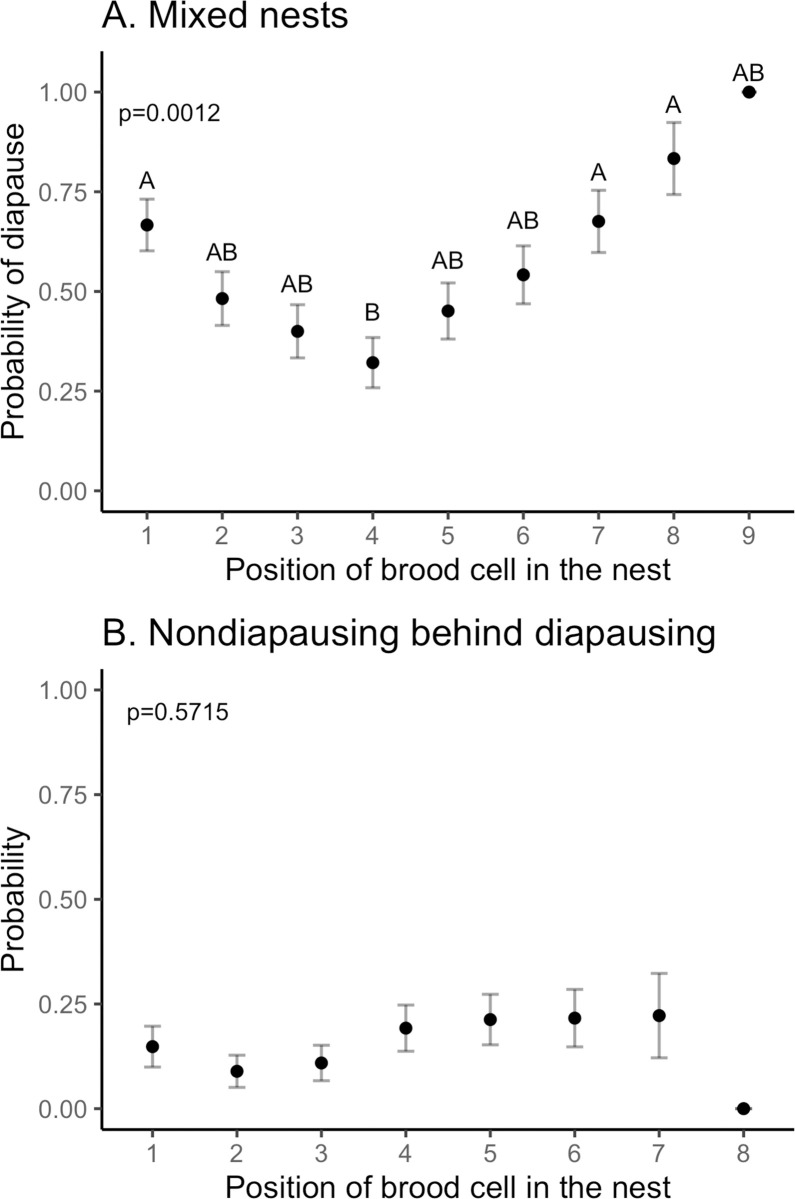
Mixed nests contained both diapausing and nondiapausing bees. (A) The probability of diapausing was significantly different by position of the brood cell in the mixed nests. Letters denote significant comparison by Tukey’s HSD. (B) The probability that a nondiapausing bee was laid behind a diapausing sibling was independent of brood cell position. Position one indicates the first brood cell built in the nest. Whiskers denote the standard error of the mean.

Some females built nests that were mixed, containing both diapausing and nondiapausing offspring. Out of 302 nests, 241 nests contained only diapausing offspring, five nests contained only nondiapausing offspring, and 56 nests were mixed with diapausing and nondiapausing offspring. Mean nest size was not significantly different between the three types of nests (ANOVA, F_(2, 299)_ = 1.126, p = 0.33). For mixed nests, the position of the brood cell in the nests significantly influenced diapause status (ANOVA; F_(8,368)_ = 3.2993, p = 0.0012), with the fourth brood cell being the most likely to contain a nondiapausing bee (Fig 4A), with nests averaging 6.8 brood cells. Diapausing offspring that are laid after nondiapausing offspring in the nest will possibly be killed by the nondiapausing offspring when they emerge. Our results suggest that nondiapausing offspring are frequently laid behind diapausing offspring (Fig 4A). We calculated the probability that a nondiapausing bee was laid behind a diapausing bee for each brood cell position (Fig 4B). That arrangement occurred for 15.8% of the brood cells across all mixed nests. The probability of a nondiapauser positioned behind a diapauser did not vary significantly by brood cell position (F_(7,313)_ = 0.8197, p = 0.57).

### 3.4 Impacts of temperature and diapause status on survival

**Fig 5:**
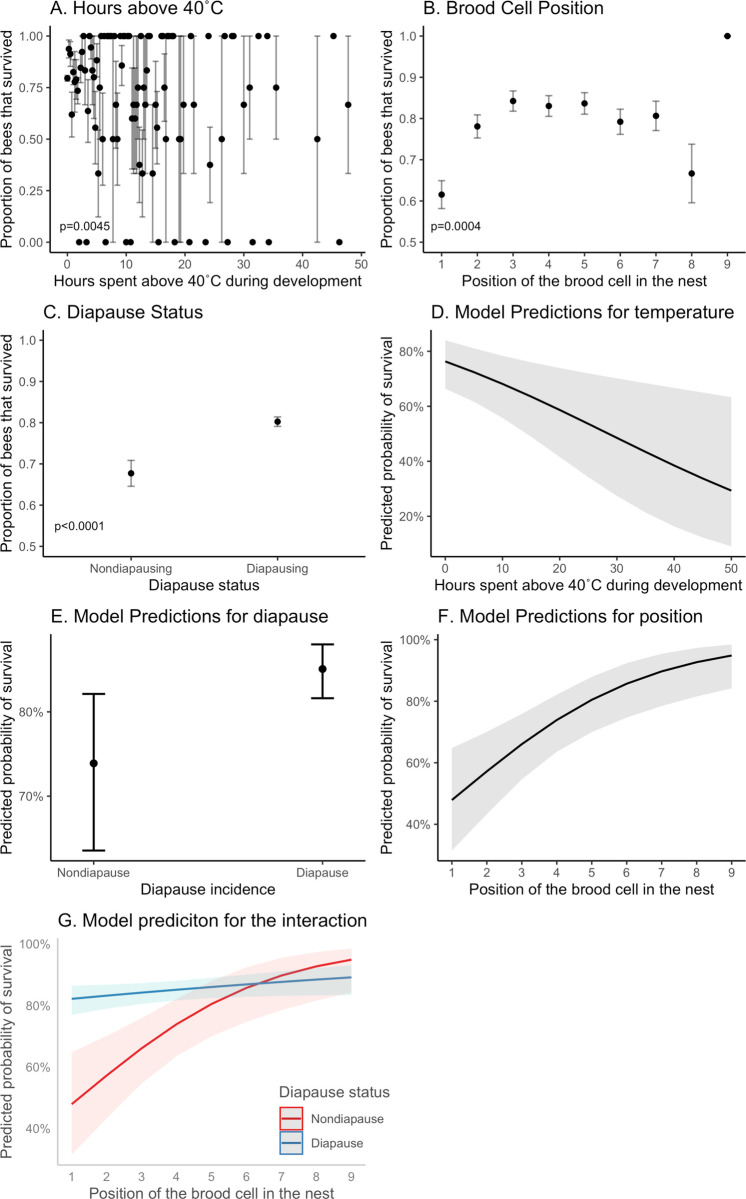
Survival to adulthood. (A) The number of hours spent above 40°C significantly influenced survival. (B) The position of the brood cell in the nest, with 1 being the first brood cell built, significantly influenced survival. (C) Whether an individual chose to diapause or not diapause was significant. The predicted probabilities from the general linear mixed model for diapause incidence (D) and brood cell position (E) reinforce these patterns and show a significant interaction between the variables (F). Whiskers on A-C denote the standard error of the mean. Grey region in D-F denotes the 95% confidence interval around the model estimates.

We determined which factors influence adult emergence, as a measure of survival to adulthood. The survival analysis included 1,428 bees from 247 nests. Most bees survived with an adult emergence rate of 78.3%. Temperatures were calculated from the date the egg was laid until 22 days later. Hours spent above 40°C significantly impacted survival (Z_(1, 1428)_ = -2.842, p = 0.0045). Diapause status (Z_(1, 1428)_ = 3.998, p<0.0001) and position of brood cell in the nest (Z_(1, 1428)_ = 3.544, p = 0.0004) also significantly impacted survival, as did their interaction (Z_(1, 1428)_ = -2.687, p = 0.0072). Daylength was not significant (Z_(1, 1428)_ = -0.719, p = 0.47). The covariates explained 5% of the variation in survival. The random effect of maternal ID increased the R^2^ to 0.29. The model that included hours above 40°C was significantly better than a model that included average temperature (delta AIC = 1, χ^2^ = 1.648, df = 0, p<0.0001). Survival decreased with an increase in the hours spent above 40°C (Fig 5A and 5D) with some bees spending over 40 hours above 40°C. Brood cells that were laid in the first and eighth position had higher mortality (Fig 5B and 5F). Nondiapausing individuals had a significantly higher mortality than diapausing individuals (Fig 5C and 5E), and this was particularly the case for nondiapausers in the first few nest positions (Fig 5F). In addition to the hours spent above 40°C, we also tested hours above 35°C (Z_(1, 1428)_ = -1.422, p = 0.16) which did not significantly impact survival.

## 4. Discussion

Even though some studies have found that both daylength and temperature can influence diapause incidence in *M*. *rotundata* [5, 10, 11], our study suggests that daylength may have a strong effect on the behavior of this bee species. We tested the relative contribution of daylength and temperature to diapause incidence under field conditions in Fargo, ND, USA. We found that daylength influenced diapause, but that nest cavity temperature did not have an effect on diapause incidence.

### 4.1 Diapause incidence

We predicted that warmer temperatures would cause higher rates of nondiapausing bees. In this study, all cavities reached 35˚C during the developmental period and many cavities spent 20 hours or more over 40˚C. The warmest cavities in our study were the southwest and southeast facing cavities which spent the most time above 35˚C and 40˚C. Despite high field temperatures, there was no effect of these temperatures on diapause incidence. Neither time spent over 35˚C, nor 40˚C influenced diapause rates. We also tested a degree day model that uses a lower developmental threshold of 18˚C and an upper threshold of 35˚C, but this variable was also not significant. Constant temperatures between 29˚C and 32˚C have been reported to increase the frequency of nondiapausing *M*. *rotundata* prepupae under lab conditions, but average temperatures of 22˚C and 26˚C did not have an effect [5]. Our average nest cavity temperatures during the developmental period ranged from 20.2–26.3˚C with a mean of 24.0˚C. These field temperatures are within the range that would not affect diapause incidence based on these previous lab studies [5, 10]. Kemp and Bosch [5] found that fluctuating temperatures had diapause rates similar to the mean of the fluctuation, not the peak temperature. Although our peak temperatures were consistently over 35˚C and often exceeded 40˚C, nighttime lows kept the average temperatures below the range warm enough to influence diapause initiation. Based on our measurement of field nest box temperatures, nest cavities would need to have mid-day peak temperatures well above 40˚C to produce average cavity temperature above 29˚C. Our measurement of field temperatures suggest that it is unlikely that nest temperature would ever be high enough to influence diapause initiation while also allowing for survival to the adult stage.

Daylength had a significant influence on diapause initiation, with eggs laid during longer days associated with higher rates of nondiapausers. Daylength decreased during the duration of the study, with the first nest capped on June 23^rd^. Daylength explained 42.8% of the variation in diapause incidence. Tepedino and Parker [36] found the majority of nondiapausing individuals are laid by July 22^nd^ and argued that daylength is a trigger for diapause initiation. All of the nondiapausing individuals in our study were laid by July 23^rd^, which follows the pattern seen in several other studies across various latitudinal rages [4, 10, 14, 36]. Exposing females to very long days can decrease diapause initiation in offspring [11]. The longest daylengths in this study correspond to the “long day” treatment in Pitts-Singer [11], but the diapause rate in this study was lower. Once we accounted for daylength, most of the remaining variation in diapause initiation was explained by the ID of the mother. The mother ID encompassed many possible sources of variation. Some of this variation may be caused by environmental cues the mother senses that were not monitored as part of this study, standing genetic variation for diapause incidence [37], and maternal epigenetic effects [38]. Mothers may mediate these cues through the amount of provision, which is larger in diapause-destined offspring [12]. In addition, the mother ID random effect includes variance caused by environmental conditions in the cavity shared by each of the brood cells in that nest, and environment conditions in the field during the point in the season when the nest was built. A future direction of this work is to test these potential sources of variation in a controlled experiment.

Some nests contained both diapausing and nondiapausing offspring. These mixed nests accounted for 18.5% of all nests, and the majority of the nondiapausing individuals were laid in mixed nests. *Megachile rotundata* females lay eggs in a linear nest with emergence patterns in the reverse order from the order the eggs are laid [4, 39, 40]. Mixed nests have the potential to increase mortality if diapausing bees have nondiapausers laid behind them. When the nondiapausing bees emerge, they have the potential to kill the diapausing bees as they chew their way to the entrance. This unfavorable arrangement occurred in 15.8% of the brood cells in mixed nests.

The cause of mixed nests remains unknown, but could be related to differences in diapause rates between males and females. We observed that nondiapausing bees are predominately female, and diapausing bees are predominately male, a pattern seen in another study [36]. The sexes are mixed within nests with some studies finding females laid toward the back [39–41]. The interaction of sex, diapause, and cell position could account for deleterious ordering of nondiapausers in mixed nest [7]. We could not directly test this hypothesis because we do not have sex information for diapausing individuals from mixed nests. We can conclude that mixed nests were common in our study and represent a potential source of mortality for diapausing bees.

### 4.2 Survival

Nest boxes can reach temperatures that can cause high mortality [15, 42, 43]. Females may avoid nesting in warmer cavities to protect offspring [15]. Temperatures exceeding 35˚C induce the production of HSP70 proteins, which is an indicator of stress [28]. Mortality is high at a constant exposure to 44˚C [44]. In this study, nest cavities frequently exceeded 40˚C, and the number of hours spent over 40˚C significantly decreased survival. All the cavities spent some time over 35˚C but that temperature threshold was not enough to cause mortality. Exposure to lower temperatures in between time spent above 35˚C may have protected bees from heat stress due to either physiological preparation for stressful exposure [45] or by allowing time to repair damage [46]. Previous research from this same field season found that females prefer to nest in cavities with cooler temperatures [15]. Females nesting later in the season were forced to choose warmer cavities because fewer cavities remained open. Wilson et al. [15] found females did not prefer south-facing cavities. The analysis conducted here determined that south-facing cavities spent the highest number of hours over 40˚C. Females choosing cavities based on cooler temperatures may protect offspring from heat stress. However, the statistical model only explained 29% of the variance in survival. Our results indicate that temperatures in nest cavities can reach lethally-high levels.

Diapause status and position of the brood cell in the nest both significantly affected survival. Nondiapausing bees had a significantly lower survival (67.7%) than diapausing (80.3%). Diapause status had a significant interaction with brood cell position such that nondiapausers positioned in the first brood cells had the lowest survival. The significance of position may be influenced by a female-biased sex ratio for nondiapausers and females frequently being laid in the beginning of each nest. Kemp and Bosch [5] reported higher mortality in diapausing bees compared to nondiapausers, which is the opposite pattern we observed. Overall, adult emergence was 78.3%, which is higher than the 40–60% mortality observed in natural populations [8, 42].

### 4.3 Limitations of the study

We observed nest supersedure, when a female destroys the brood cells of another to build her own. Supersedure was easily identifiable with either a larva sitting on the base of the board completely exposed to the elements, or female bees were observed actively removing leaf pieces from pre-existing cavities. Any nest that changed in size from the initial to the final x-ray was removed from analysis. This decreased our sample size and may have reduced statistical power in the analysis of diapause because the majority of superseded nests were in built under long daylengths and likely contained a higher proportion of nondiapausers. If more nondiapausers had been present in the dataset, that may have increased the statistical power to detect the effects of other factors such as temperature.

Another limitation of our study comes from the mixed nests that contained diapause and non-diapause bees. Individuals from mixed nests that were diapausing were sacrificed from the straws in order to follow the nondiapause individuals. Thus, we do not know the sex of these sacrificed bees. From the sex of the remaining bees and from previous studies, diapausing and nondiapausing bees have different sex-ratios with diapausing bees being male-biased [36]. However, we could not incorporate sex into our statistical models for diapause incidence because diapausing bees in mixed nest had unknown sex and those individuals were biased to be in the front of the nest. This suggests that many of the sacrificed bees would have been probably male [40].

### 4.4 Conclusions

We established that daylength had a strong influence on the diapause status of *M*. *rotundata*. Nest cavity temperature did not influence diapause over the ranges measured in this field study, and average nest temperatures during development remained lower than the temperatures known to influence diapause from lab-based studies. Maternal factors influence diapause, as established by daylength explaining a lot of the variation in diapause incidence. Mixed nests containing both diapausing and nondiapausing individuals were common. Under agricultural management, mixed nest represent a potential loss to the yield of diapausing bees. Nest cavity temperatures were high enough to increase mortality but not influence diapause incidence, suggesting that the main threat to agricultural populations under climate change may be a loss of field yield due to heat-stress, not a decrease in rates of diapause.
